# Morphometrics Parallel Genetics in a Newly Discovered and Endangered Taxon of Galápagos Tortoise

**DOI:** 10.1371/journal.pone.0006272

**Published:** 2009-07-17

**Authors:** Ylenia Chiari, Chaz Hyseni, Tom H. Fritts, Scott Glaberman, Cruz Marquez, James P. Gibbs, Julien Claude, Adalgisa Caccone

**Affiliations:** 1 Department of Ecology and Evolutionary Biology and YIBS-Molecular Systematics and Conservation Genetics Lab, Yale University, New Haven, Connecticut, United States of America; 2 Museum of Southwestern Biology, University of New Mexico, Albuquerque, New Mexico, United States of America; 3 Postal del Parque Nacional Galápagos, Galápagos, Ecuador; 4 College of Environmental Science and Forestry, State University of New York, Syracuse, New York, United States of America; 5 Institut des Sciences de l'Evolution, CNRS-UMR, Université Montpellier 2, Montpellier, France; University of Otago, New Zealand

## Abstract

Galápagos tortoises represent the only surviving lineage of giant tortoises that exhibit two different types of shell morphology. The taxonomy of Galápagos tortoises was initially based mainly on diagnostic morphological characters of the shell, but has been clarified by molecular studies indicating that most islands harbor monophyletic lineages, with the exception of Isabela and Santa Cruz. On Santa Cruz there is strong genetic differentiation between the two tortoise populations (Cerro Fatal and La Reserva) exhibiting domed shell morphology. Here we integrate nuclear microsatellite and mitochondrial data with statistical analyses of shell shape morphology to evaluate whether the genetic distinction and variability of the two domed tortoise populations is paralleled by differences in shell shape. Based on our results, morphometric analyses support the genetic distinction of the two populations and also reveal that the level of genetic variation is associated with morphological shell shape variation in both populations. The Cerro Fatal population possesses lower levels of morphological and genetic variation compared to the La Reserva population. Because the turtle shell is a complex heritable trait, our results suggest that, for the Cerro Fatal population, non-neutral loci have probably experienced a parallel decrease in variability as that observed for the genetic data.

## Introduction

Conservation biologists rely on systematics to properly recognize taxonomic units in order to protect them (e.g., [1]). Since the time of Linnaeus, taxonomic classification has largely been based on morphological characters. However, morphology alone can sometimes be misleading. High intraspecific phenotypic variation can be mistaken as evidence of multiple species (e.g., [2]), while legitimately separate species can be improperly combined due to similarities in morphology (e.g., [3]). The integration of morphological and genetic information has increasingly been used to resolve such uncertainties.

Perhaps equally as important, combining different types of data can also make it possible to indirectly infer the vulnerability of a population facing environmental disturbance. Genetic and/or phenotypic variation can reflect the capacity of a population to respond to different types and levels of stress since it is the raw material upon which adaptation can take place (e.g., [4]). However, conservation measures based on genetic data frequently reflect the analysis of genetic markers that are not necessary subject to natural selection [5]. In addition, phenotypic variation does not always reflect the observed genetic diversity of a population. This is due to the lack of association between the genes analyzed and traits that are easily measurable (reviewed in [6]), and to the fact that phenotypic variation is partly under the control of non-additive genetic variation. Thus, while neutral markers may serve as one measure of the genetic impact of stress on a population (e.g., low genetic variation, bottleneck), the examination of phenotypic traits that show high heritability could be used as a proxy to evaluate the level of genetic variation at non-neutral loci within a population.

Galápagos tortoises are an emblematic and important taxon about which little is currently known and thus additional work is greatly required. These animals are in various stages of endangerment [7], [8], and they possess numerous characteristics that are often associated with greater risk of extinction [9], [10], including island endemism, slow growth rate, late sexual maturity, and large body size. The distinctiveness of extant Galápagos tortoise lineages (11 currently recognized taxa inhabiting six islands) was initially based mostly on diagnostic morphological characters of the shell [11]. More recently, molecular studies revealed that each island harbors a distinct monophyletic lineage, with the exception of Isabela and Santa Cruz, where multiple lineages have been documented [3], [12], [13], [14], [15].

Santa Cruz is one of the islands in the Galápagos archipelago that has been most strongly impacted by human disturbance. As a consequence, the range of tortoises on the island has been reduced. Only a single taxon (*Geochelone nigra porteri* or *G. porteri*, but see also [16]) is currently recognized on the island; however, three genetically distinct tortoise lineages have been shown to inhabit Santa Cruz [3], [14]. Each of these three lineages has a sister taxon on nearby islands from which they are genetically highly divergent at the mitochondrial and nuclear level [12], [13]. Two of these lineages exhibit a general domed morphology, while the third, which is probably composed of only a few individuals at present, possesses the saddleback morphology (see [17] for a description of the domed and saddleback morphologies). The two domed tortoise populations, referred to as La Reserva and Cerro Fatal, differ in their geographic distribution, population size, and level of genetic diversity [3], [7], [14]. Despite a lack of visible morphological differentiation, the two domed lineages are as genetically distinct from each other as from tortoises occurring on other islands. In fact, they occupy clades that are reciprocally monophyletic and linked to each other through the deepest node of the Galápagos tortoise phylogeny [3]. While the La Reserva population harbors one of the largest tortoise population in the archipelago (ca. 2000–3000 individuals), the Cerro Fatal population has a much smaller population size and recently experienced a strong population decline due to human habitat disturbance and heavy poaching. Only about 100 individuals were estimated to have existed in 1974 [7], and this is likely the cause of the dramatically low levels of genetic diversity at mitochondrial and nuclear microsatellite loci that are currently found in this population [3].

In turtles, genetic and morphometric data have often been combined to resolve taxonomic uncertainties (e.g., [18], [19], [20]). The first objective of the current study is to integrate the two types of data in order to evaluate whether the genetic distinctiveness of the two domed Santa Cruz tortoise populations is also paralleled by morphological differences in shell shape. Corroborative data could provide broader and more comprehensive support for the distinctiveness of the two domed populations on the island. We also investigate whether genetic variation at neutral loci is correlated with morphological variation within each population in order to test whether Cerro Fatal lost genetic variation underlying quantitative traits as well. Here, we integrate nuclear and mitochondrial data with statistical analysis of shell shape morphology using linear and curved measurements. We chose to focus on shell variation because measurements can be precisely collected on this structure and because of its complexity as a morphological trait, resulting from the interaction of many genes [21].

## Results

### Morphometrics

Table 1 shows the different mean size for each populations and sex. The two populations differed in mean size and mean shape (Pop as factor, Table 2 and Table 3). Within the two populations, the sexes were dimorphic in size and mean shape, but since the interactions (Sex x Pop) were not statistically significant, sexual dimorphism is expressed in a similar way in both populations (Table 2 and Table 3). Populations did not differ in their allometric coefficient (for allometric coefficient see [22]) (Log(size) x Pop, Table 4), however they differed in mean shape (Pop, Table 4). This means that shape differences between populations are preserved and correspond to different shape proportions during growth. Conversely, sexes showed differences in allometric coefficients (Log(size) x Sex, Table 4) and were found to be similar in mean shape once allometry was filtered out (Sex, Table 4). This means that differences between males and females accumulate during growth, with males having higher values of allometric coefficients than females (data not shown).

**Table 1:** Mean size.

Population	Sex	Sample size	Mean geometric size (mm)	Standard deviation
**Cerro Fatal**		32	257.5022	59.00279
	**Males**	17	290.2313	50.09574
	**Females**	15	220.4093	45.44959
**La Reserva**		49	286.6327	64.06705
	**Males**	21	325.1621	73.22504
	**Females**	28	257.7357	36.12028

Mean size of tortoises from La Reserva and Cerro Fatal populations when grouped by population and sex.

**Table 2:** ANOVA on mean size differences.

Effect	*df*	Mean Squares	F	P-value
Sex	1	93377	33.9894	10^−07^ *
Pop	1	25044	9.1162	0.003*
Sex x Pop	1	27	0.0100	0.920
Error term	77	2747		

Mean size distinction between tortoises from La Reserva and Cerro Fatal populations. Two-way ANOVA on size with population (Pop) and sex (Sex) as factors.

* indicates significant p-value (p<0.05).

x indicates the interaction between factors. *df* = degree of freedom.

**Table 3:** Shell shape differences.

Effect	*df*	Hotelling-Lawley trace	Approx F	*df* num	*df* den	P-value
Sex	1	1.1296	2.2592	26	52	0.006*
Pop	1	3.1474	6.2948	26	52	10^−08^ *
Sex x Pop	1	0.5851	1.1701	26	52	0.308
Error term	77					

Shell shape distinction between tortoises from La Reserva and Cerro Fatal populations. Two-way MANOVA on shape variables with population (Pop) and sex (Sex) as factors.

* indicates significant p-value (p<0.05).

x indicates the interaction between factors. *df* = degree of freedom. *df* num = degree of freedom numerator. *df* den = degree of freedom denominator.

**Table 4:** Shell shape differences once allometric growth is removed.

Effect	*df*	Hotelling-Lawley trace	Approx F	*df* num	*df* den	P-value
Sex	1	0.9947	2.2057	23	51	0.107
Pop	1	3.1485	6.9815	23	51	2×10^−08^ *
Log(size) x Pop	1	0.6343	1.4065	23	51	0.052
Log(size) x Sex	1	0.6.744	1.4954	23	51	0.038*
Sex x Pop	1	0.4950	1.0977	23	51	0.380
Log(size) x Pop x Size	1	0.4034	0.8944	23	51	0.604
Error term	73					

Shell shape distinction between tortoises from La Reserva and Cerro Fatal populations once allometric growth is filtered out. Multivariate analysis of covariance taking into account Log(size) as covariate, and sex (Sex) and population (Pop) as factors.

* indicates significant p-value (p<0.05).

x indicates the interaction between factors. *df* = degree of freedom. *df* num = degree of freedom numerator. *df* den = degree of freedom denominator.

**Figure 1:**
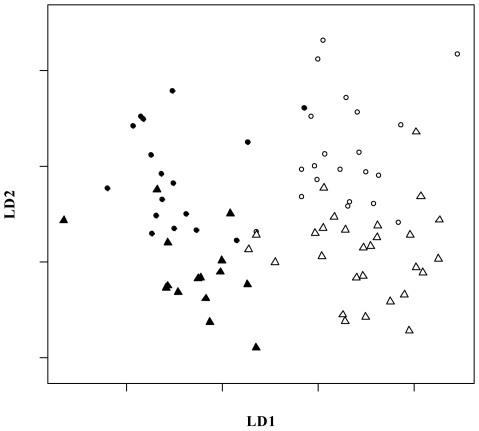
Graph of the linear discriminant analysis. Linear discriminant analysis along the first and second discriminant axes (LD1 and LD2, respectively). LD1 and LD2 account for 66% and 22% discriminant power, respectively. Black circles = Cerro Fatal males. White circles = La Reserva males. Black triangles = Cerro Fatal females. White triangles = La Reserva females.

Figure 1 shows the results of the linear discriminant analysis. The two populations and sexes separate along the first and second discriminant axes, respectively. The percentage of discriminant power associated with the first axis was 66%, while the one associated with the second axis was 22%. Tortoises from La Reserva had a slightly flatter and more elongated carapace with a slightly higher anterior opening, thus exhibiting a slight tendency toward a saddleback morphology compared to tortoises from Cerro Fatal. Moreover, the sexual dimorphism was characterized by the females of each population being less domed and with slightly longer and wider carapaces than males (table of discriminant coefficients not shown).

Table 5 shows the estimated size and shape variation of tortoises from the La Reserva and Cerro Fatal populations (Table 5). La Reserva tortoises have larger variation in size and shape compared to the Cerro Fatal population (Table 5). The difference in size variation between the two populations was not statistically significant (Table 6). The difference in shape variation between both populations was not statistically significant (Table 7), but became significant once variation due to allometric growth was removed (Table 8). This suggests that the differences in shape variation between populations were not due to sampling from different growth stages in each population. In both populations, males varied more in size and shape than females (Table 5), but differences in size variation between sexes within each population were significant only for the La Reserva population (Table 6). Differences in shape variation between sexes were significant within each population (Table 7), but were not statistically significant once variation due to allometry was removed (Table 8). This indicates that differences in raw shape variation between sexes are probably due to allometric differences between males and females and to the different growth stage of the individuals sampled for each sex. The fact that shell variation was better explained by growth for males than for females (Cerro Fatal 42% in males and 17% in females; La Reserva 29% in males and 8% in females) further supports this conclusion.

**Table 5:** Size and shape variation.

Population	Sex	Size variation	Shape variation	Shape variation (no allometric growth)
**Cerro Fatal**		3481.329	0.125	0.092
	**Males**	2509.583	0.135	0.083
	**Females**	2065.665	0.086	0.081
**La Reserva**		4104.587	0.139	0.120
	**Males**	5361.906	0.167	0.123
	**Females**	1304.674	0.109	0.109

Total level of variation in size, shape, and shape with allometric growth filtered out for tortoises from La Reserva and Cerro Fatal populations grouped by population and sex.

**Table 6:** Size variation differences.

Comparisons	Var. obs	Sample size 1	Sample size 2	P-value
Cerro Fatal vs La Reserva	1.179	32	49	0.271
Cerro Fatal M vs F	1.2149	17	15	0.307
La Reserva M vs F	4.1098	21	28	0.000*
Cerro Fatal M vs La Reserva M	2.1366	17	21	0.008*
Cerro Fatal M vs La Reserva F	1.9235	17	28	0.053
Cerro Fatal F vs La Reserva F	1.5833	15	28	0.086
Cerro Fatal F vs La Reserva M	2.5957	15	21	7×10^−04^ *

Exact test on the level of size variation between the different studied groups (tortoises organized by population and sex).

* indicates significant p-value (p<0.05).

M = males. F = females. Var. obs. = observed variance. “Sample size 1” and “Sample size 2” refer to the order of the comparison (first columns on the left of the table).

**Table 7:** Shape variation differences.

Comparisons	Var. obs.	Sample Size 1	Sample size 2	P-value
Cerro Fatal vs La Reserva	1.112109	32	49	0.185
Cerro Fatal M vs F	1.568833	17	15	0.043*
La Reserva M vs F	1.529417	21	28	0.001*
Cerro Fatal M vs La Reserva M	1.238719	17	21	0.060
Cerro Fatal M vs La Reserva F	1.234676	17	28	0.101
Cerro Fatal F vs La Reserva F	1.270643	15	28	0.034*
Cerro Fatal F vs La Reserva M	1.943343	15	21	10^−4^ *

Exact test on the level of shape variation between the different studied groups (tortoises organized by population and sex).

* indicates significant p-value (p<0.05).

M = males. F = females. Var. obs. = observed variance. “Sample size 1” and “Sample size 2” refer to the order of the comparison (first columns on the left of the table).

**Table 8:** Shape variation differences once allometric growth is removed.

Comparisons	Var. obs.	Sample size 1	Sample size 2	P-value
Cerro Fatal vs La Reserva	1.298232	32	49	0.013*
Cerro Fatal M vs F	1.025196	17	15	0.449
La Reserva M vs F	1.122052	21	28	0.216
Cerro Fatal M vs La Reserva M	1.482422	17	21	0.021*
Cerro Fatal M vs La Reserva F	1.321171	17	28	0.039*
Cerro Fatal F vs La Reserva F	1.354460	15	28	0.026*
Cerro Fatal F vs La Reserva M	1.519774	15	21	0.015*

Exact test on the level of shape variation between the different studied groups (tortoises organized by population and sex) once allometric growth is filtered out.

* indicates significant p-value (p<0.05).

M = males. F = females. Var. obs. = observed variance. “Sample size 1” and “Sample size 2” refer to the order of the comparison (first columns on the left of the table).

### Genetics

The newly collected microsatellite data were combined with the ones from 136 individuals previously analyzed at the same loci [3], [13], [14], resulting in a total of 236 individuals available for microsatellite analysis (115 from Cerro Fatal and 121 from La Reserva). Out of the 100 new DNA samples that were collected, mtDNA sequence data for the control region (690 bp) was obtained for 96 individuals. These data were combined with the previously available 128 sequences for the same marker (65 from Cerro Fatal and 63 from La Reserva; [3], [14]) for a total of 224 individuals available for mitochondrial analysis.

Levels of microsatellite variability were substantially higher in the La Reserva population than in the Cerro Fatal population (Table 9). The highest number of alleles at a single locus was 32 in La Reserva compared to only nine in Cerro Fatal. The mean number of alleles across all nine loci was 17.2 and 5.3 in La Reserva and Cerro Fatal, respectively, while mean expected heterozygosity (H_E_) was 0.81 and 0.58 in the two populations, respectively. Departure from Hardy-Weinberg equilibrium was observed for seven of the nine loci in the La Reserva and six loci in the Cerro Fatal population (p<0.05; four and five respectively after Bonferroni correction for multiple tests). In La Reserva, this departure from Hardy-Weinberg equilibrium is due to heterozygote deficiency (six loci, p<0.05; four after Bonferroni correction); in Cerro Fatal heterozygote excess was recorded for three loci (p<0.05; one after Bonferroni correction). Linkage disequilibrium was detected in both populations indicating a non-random association between loci in 10 pairwise comparisons in La Reserva and 29 in Cerro Fatal (p<0.05). The sequential Bonferroni corrections within populations reduced the number of significant non-random associations to one for La Reserva and 16 for Cerro Fatal.

**Table 9:** Genetic diversity based on nine microsatellite loci.

Microsatellites						
Population	N	N. of Loci		N. of alleles	H_E_	H_O_
**Cerro Fatal**	115	9	**Mean±SD**	5.33±2.55	0.58±0.15	0.61±0.17
**La Reserva**	121	9	**Mean±SD**	17.22±9.76	0.81±0.16	0.75±0.17

Measures of genetic diversity for the Cerro Fatal and La Reserva Galápagos tortoise populations based on nine microsatellite loci. N = number of individuals analyzed. H_E_ = expected heterozygosity. H_O_ = observed heterozygosity. SD = standard deviation.

When individuals were assigned to populations to define their ancestry, the analysis delimits two clusters in the dataset (most likely value of *K* = 2). More than 90% of the tortoises in both populations were correctly assigned to their original population. Individuals sampled in Cerro Fatal were assigned to their cluster with an average proportion of membership of 0.99, while for La Reserva the coefficient was 0.96. Additionally this analysis detected seven individuals of potential mixed origin between these two populations.

The 224 mtDNA sequences resulted in 26 distinct haplotypes. Twenty-one haplotypes were found in La Reserva, while the other five haplotypes were found in Cerro Fatal. The Cerro Fatal and La Reserva haplotypes grouped into two highly distinct haplotype networks, separated by 28 mutational steps (Figure S1, supporting information). However, one haplotype, connected to the rest of the Cerro Fatal haplotype network by five mutations, belonged to an individual sampled in La Reserva (Figure S1, supporting information). The Cerro Fatal haplotype network is dominated by one major haplotype presents at a frequency of 83%. Genetic diversity at the mitochondrial level in La Reserva, on the other hand, was more structured, with only a few of the 21 haplotypes found at a frequency higher than 5%. The tortoises in the two populations also differed considerably in levels of mitochondrial haplotype diversity, *h* (Cerro Fatal = 0.30, La Reserva = 0.85, Table 10). The AMOVA of the control region sequences revealed that most of the variation (90%) is due to between-population differences (within population difference only 10%, p<0.0001). The existence of strong genetic differentiation between the two populations was confirmed by mitochondrial and microsatellite fixation indices (F_ST_ = 0.897 and θ = 0.148, respectively, p<0.0001).

**Table 10:** Genetic diversity based on the mitochondrial control region.

Mitochondrial DNA				
Population	N	N. of haplotypes		*H*
**Cerro Fatal**	107	5	**Mean±SD**	0.30±0.05
**La Reserva**	117	21	**Mean±SD**	0.85±0.02

Measures of genetic diversity for the Cerro Fatal and La Reserva Galápagos tortoise populations based on a 690 bp fragment of the mtDNA control region. N = number of individuals analyzed. *h* = haplotypic diversity. SD = standard deviation.

## Discussion

The shell morphometric analyses parallel the genetic distinctiveness found between the two Galápagos tortoise populations on Santa Cruz. The two populations differ in size (Table 1 and Table 2), with the tortoises from La Reserva being bigger than those from Cerro Fatal. Shape also differed (Table 3), with tortoises from Cerro Fatal being slightly more domed than tortoises from La Reserva (data not shown). Moreover, differences in shape between populations are not related to different allometric growth patterns (Table 4), meaning that if the populations were sampled at the same growth stage they would still differ in their shell shape. A combination of morphometric variables from the plastron and carapace were able to discriminate between the two populations as well as between sexes (Figure 1).

Mitochondrial and nuclear data show that the two Galápagos tortoise populations on Santa Cruz are highly distinct, despite a few individuals being identified as hybrids. Species recognition and range boundaries are difficult to recognize in rapidly speciating taxa (as reviewed in [23]), mainly due to hybridization and rapid morphological divergence. Thus, the prevailing question requiring further deliberation is whether the two studied populations represent distinct species or reflect adaptive variation within the same species and hence separate units under the adaptive evolutionary concept (ACE, [24]; see discussion in [25] for a background on evolutionary units and species concepts). A hybrid zone between the two populations is currently not known. Moreover, hybridization occurs at low rates in the wild and in captivity in Galápagos tortoises when individuals of distinct ancestry meet (e.g., [26]). Our results show that individuals of mixed origin between the two populations are rare (only 3% of all sampled individuals), possibly resulting from the migration and consequent hybridization of a very small set of individuals between populations or the greater proximity of the taxa's historical ranges (prior to current settlement of the agricultural zone between them that likely extirpated tortoises from the area). Whatever the case, nuclear and mitochondrial genetic distances between the two populations are comparable to the genetic distances existing among recognized distinct lineages of Galápagos tortoises (see [12] for mitochondrial absolute distances and [13] for microsatellite distances).

Neither shell morphology (saddleback and domed) nor island of origin are reliable for distinguishing among taxa of Galápagos tortoises. For example, domed and saddleback shell forms seem to have evolved multiple times in the archipelago [27]. In the same way, based on recent data, distinct evolutionary lineages exist on the same island (as in Santa Cruz and Isabela, [3], [12], [13], [14], [15]). However, the current taxonomy of the group remains a source of debate, with distinct lineages indicated either as one species or distinct species or subspecies (e.g., [27], [28], [29], [30]). The two Galápagos tortoise populations on the island of Santa Cruz are currently described as one single species due to their similar shell morphologies (both domed) and the fact that they occur on the same island. However, genetic distances at nuclear and mitochondrial levels, as well as morphological differences, indicate the existence of two separate evolutionary lineages on this island. In particular, the genetic distances between the two populations are comparable to the ones of separate evolutionary lineages inhabiting different islands (and indicated as separate species in [27]). Thus, the two populations represent at least distinct evolutionary and conservation units under the adaptive evolutionary concept, which is a more integrated and flexible concept than the ESU [31], [32], taking into account not only genetic distances at mitochondrial and nuclear markers, but also other differences characteristic of each evolutionary unit (e.g., shell morphology). Our results further support the need for taxonomic revision of Galápagos tortoises based on the integration of different datasets (genetic diversity and shell morphology differences within and among lineages) that have yet to be generated.

Our results additionally suggest that the amount of variation in shell shape is different in these two populations, which also parallels the genetic diversity results. Cerro Fatal shows heterozygote excess, high levels of linkage disequilibrium (both of which can be explained by a past bottleneck), and much less genetic variability overall at mitochondrial and nuclear loci than the La Reserva population (Table 9 and Table 10 and [3], [13], [14]). The low genetic diversity observed in Cerro Fatal tortoises has been suggested to have resulted from the more recent founding of this population by migrants from another island, San Cristóbal [3], [12], and a population size reduction due to human disturbance [7]. On the other hand, the La Reserva population is one of the largest and most genetically diverse tortoise populations in the Galápagos [3], [13], [14].

The tortoise shell is a complex polygenic morphological trait (reviewed in [21]) that serves a variety of functions besides providing physical protection. It is important for animal self-righting [33], thermoregulation, locomotor performance [34], physiological functions such as serving as a reservoir for water, fat and wastes, and successful mating and reproduction. Thus, the tortoise shell is considered to be an important trait for individual survivorship and fitness. Myers and colleagues [35] found plastron shape variation to be highly heritable (see Table 1 [35] for heritability values), suggesting a similar heritable genetic component also for the shell. Based on this, our study suggests that non-neutral genes such as the ones involved in shell development likely also experienced a decrease in variability, as did neutral (mitochondrial control region and microsatellites) markers in the Cerro Fatal population in comparison to La Reserva.

Although the parallel genetic and morphological patterns implicate demography as a primary force in shaping both neutral and non-neutral genes frequencies, past studies have revealed a complex relationship between neutral markers and morphological characters in other recently diverged organisms (e.g., Darwin's finches, [36], [37]; sticklebacks, [38], [39]; cichlids, [40], [41]). These studies suggest that selection and plasticity may also play a role in shaping phenotypic variation and differentiation, and we therefore must consider them in the interpretation of the data. For example, in our study, while the parallel between genetic and shell shape variation between these two lineages can be explained by the different demographic histories of the two populations, the larger difference in genetic variation compared to morphological shell shape variation observed between La Reserva and Cerro Fatal tortoises (ratio of three-four times for genetics, depending if we consider the number of alleles/haplotypes or the haplotype diversity, vs. a ratio of 1.3 times for shell shape variation) requires some additional explanation. A slower rate of morphological evolution compared to molecular evolution could explain the observed pattern (but see below). However, the lack of knowledge about the amount of variation at quantitative traits involved in shell morphology, the heritability of these traits, and the influence of plasticity are all factors that impede our ability to further explore the imperfect parallelism between morphological and genetic variation. For example, it is known that diverse movement patterns and environmental diversity are known to cause dissimilarity in shell shape in other chelonians (e.g., [42], [43]). Therefore, phenotypic plasticity could explain why the relationship between neutral genes and morphology is not linear.

Natural selection may also be acting on shell morphology and may explain why the genetic divergence between the two Santa Cruz populations is much more pronounced than for morphology. If we assume that both populations are derived from a domed shell ancestor, then it would seem that stabilizing selection is acting on genes underlying shell shape. In fact, mitochondrial phylogenetic trees indicate that the two highly divergent carapace morphologies (saddleback and domed) evolved multiple times in the archipelago (e.g., [3], [12], [27]), suggesting that highly divergent shell forms could have evolved between these two populations. The large genetic divergence between the two Santa Cruz populations is in line with the timeframe in which saddleback morphology has arisen in other tortoise populations. Therefore stabilizing selection rather than a slower rate of shell evolution is more plausible. On the other hand, if either or both of the populations derive from a saddleback ancestor, then strong positive selection and convergence would have to have occurred to result in two similarly domed populations. However, the current phylogenetic data available are insufficient to hypothesize the ancestral morphology of each lineage to distinguish among the different scenarios analyzed above.

Our data also support the existence of sexual dimorphism in both populations studied on Santa Cruz. Sexual dimorphism has been observed for other populations of Galápagos tortoises [17] and it has been widely studied in chelonians ([43] and references therein). Our data indicate that sexual dimorphism occurs in a similar way within the two populations in terms of size (Table 1 and Table 2), with males being larger in size than females, as well as in terms of shape (Table 3). Moreover, independent of the population, males have increased variation in shell shape when compared to females (Table 5 and Table 7) due to a “hypermorphic” growth, which allow males to have more diverse shapes during growth. Sexual dimorphism in both populations is strongly affected by allometric growth (Table 4 and Table 8). In fact, once variation due to allometric effects is removed, shape differences between females and males of the same population disappear, indicating that these differences could be explained by the different growth stage (probably older) of males compared to females.

Based on our data, the two domed tortoise populations of Santa Cruz are genetically and morphologically distinct. The recognition of a separate taxon for the Cerro Fatal population is of primary importance for conservation and would reflect our current understanding of the evolutionary history of this group. The number of surviving individuals is low although not well defined. As our data suggest, the size and mean shape of this population, as well as the level of quantitative variation (using the shell as a proxy of a quantitative trait), parallel the extremely low genetic diversity at neutral loci, indicating a possibly reduced potential to respond to environmental disturbance.

Additional ecological and behavioral data on these populations, as well as applying a combination of morphometric and genetic analyses to other turtle populations would help to further our understanding of the relationship between shell shape variation and genetic diversity at neutral loci. Moreover, since the Cerro Fatal population is small and contains relatively few reproducing individuals producing most of the recruits, it may offer a rare opportunity to establish pedigrees in order to better understand how shell shape variation is heritable. This would offer insights into the evolution of Galápagos tortoises and their shell forms, as well as improve conservation efforts. If shell shape variation would prove to be highly heritable, then quantifying additive genetic variation could be used to identify especially endangered populations of Galápagos tortoises with direct implications for the management of these animals.

## Materials and Methods

### Ethics Statement

Animal procedures were carried out in this study following the ethics guidelines on animal handling as required by Yale University.

### Sampling

**Figure 2:**
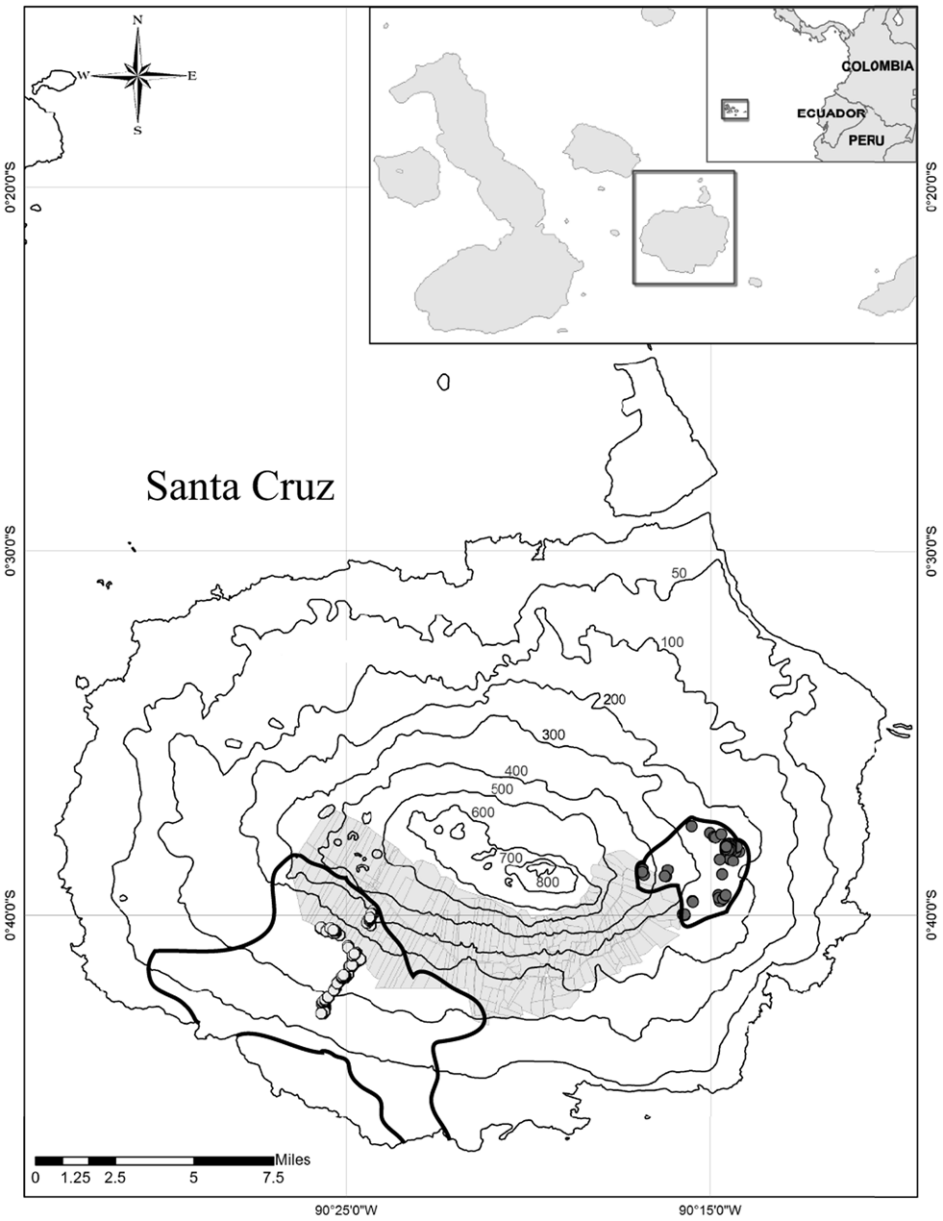
Map of Santa Cruz Island with sampling localities highlighted. Map insets indicate the geographic location of the Galápagos archipelago, west of the Ecuadorian coast, as well as the location of Santa Cruz in the archipelago. Altitude is shown on the map in meters. The shaded area represents the agricultural zone. Grey (Cerro Fatal) and white (La Reserva) circles denote samples used for the genetic analysis (including the subset of samples used for morphometric analysis, see Materials and Methods). Thicker black lines on Santa Cruz indicate the known distribution area of each of the studied populations.

Fieldwork was carried out in August 2006 on Santa Cruz Island in the Galápagos archipelago (Figure 2). We sampled a total of 122 Galápagos tortoises (64 and 58 from the Cerro Fatal and La Reserva populations respectively) in the known distribution areas of adult individuals of the two lineages. The two populations are endemic to the island of Santa Cruz and do not overlap in their distribution areas, which are currently separated by agricultural zones (Figure 2). Within the larger distribution area of the La Reserva population, preliminary data show that male tortoises occur in different areas depending on the two different seasons (hot, from December to May and cold, from June to November) [44]. The lower altitudinal range of the distribution area of La Reserva (Figure 2) is mostly occupied by juveniles [44] and it was not sampled in our study. Both populations occur and traverse very similar ecological gradients, from low, hot and dry environments to high, cool and moist ones (pers. obs.).

Geographic coordinates and elevation above sea level were recorded for each sampled individual and its sex was determined based on external features (concave plastron and characteristics of the anal scutes and the tail for males) as described in [11]. Morphological measurements and blood samples for genetic analysis were collected from individual animals, which were subsequently released. Blood was sampled only from animals that had not been previously sampled for blood in other expeditions. This resulted in a total of 100 total individuals; 45 of which were from Cerro Fatal and 55 from La Reserva. Since all sampled animals were marked with identification tags, each sample is known to represent a distinct individual. Blood samples were collected and preserved in a solution of 0.1 M Tris buffer, 0.1 M EDTA, and 2% SDS at pH 8.0 and were kept at room temperature for the duration of the field trip (four weeks) and subsequently stored at −80°C.

### Morphometrics

**Figure 3:**
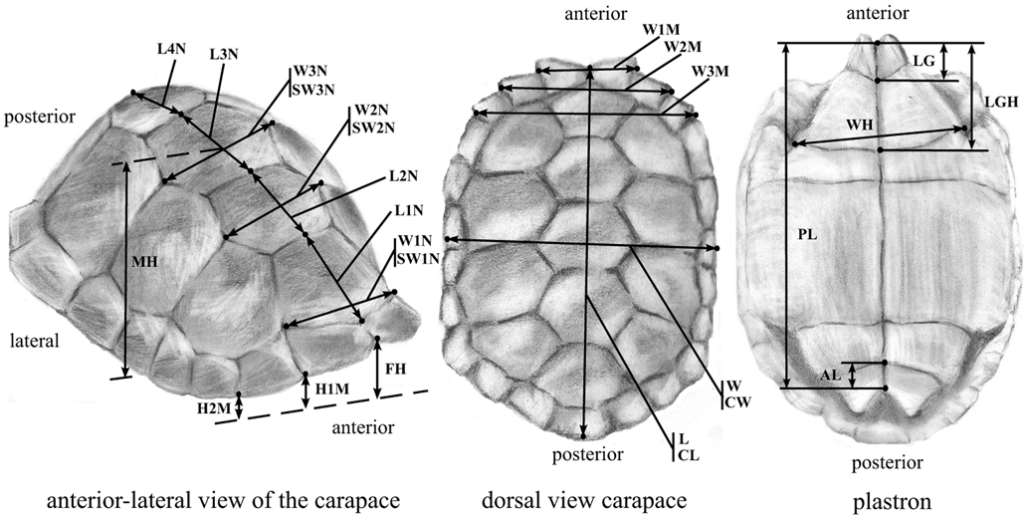
Measurements obtained on the shell. Carapace (left and center) and plastron (right) of a Galápagos tortoise with the measurements used for this study indicated (see Supporting Information S1 for details on how measurements were taken).

Measurements of 26 characters from the shell (Figure 3 and Supporting Information S1) were obtained using tree and smaller dial calipers (resolution 1.0 mm and 0.1 mm, respectively) and a flexible tape ruler (resolution 1.0 mm) for straight and curved measurements, respectively (measurements available upon request to the authors). In our analyses, we included only sexually mature individuals with a curved carapace length measuring 580 mm and above (CL, Supporting Information S1). Individuals with major injuries or shell deformities were excluded from analyses. Seven individuals of mixed origin were removed from the morphometric analyses. The final dataset consisted of a total of 32 individuals (17 males and 15 females) from the Cerro Fatal population and 49 individuals (21 males and 28 females) from La Reserva.

All morphometric analyses were run in the R environment [45] following the general framework of Claude [46]. Since the goal of morphometric analyses is to deconstruct the form of the studied object into its size and various shape components, analyses were run taking into account both size and shape variation of shell measurements independently. Size was estimated as the geometric mean from all the measurements of each individual. Shape was estimated as the original measurements divided by size (as defined above) for each individual (see [46], [47] for similar applications).

To test whether populations and sexes differ in size, a two-way analysis of variance (ANOVA) was run on size data using the F-test and type II sum of squares (factors are unbalanced within each category, see [48]) with sex and population as factors. Differences in shape between the two populations and sexes were estimated for each of the shape variables through a two-way multivariate analysis of variance (MANOVA) using the Hotelling-Lawley statistics and the type II sum of squares and cross products, also with sex and population as factors. To estimate whether sexual dimorphism was similar or different between populations, the interaction between factors was also taken into account. A linear discriminant analysis was applied using the four groups (the two different populations subdivided by the different sexes).

We also tested whether allometric growth was similar between populations and between sexes. To do this, we first log-transformed shape variables and size and then regressed the shape variables on size, taking into account the factors of population, sex, and their interaction. We then applied a multiple multivariate analysis of covariance (MANCOVA) to test if there were differences in allometric growth between populations and sexes within each population. In doing so, we also checked whether populations and sexes differed in mean shape considering allometry (e.g., whether populations differed for a given growth stage). This allowed biases introduced by the sampled individuals of each population and sex at a different growth stages to be accounted for.

Levels of shape variation were recorded as the sum of the variance of each shape variable. Although it ignores covariances, we selected this statistics because it considers that all shape variables additively account for the overall shape variation. Levels of shape and size variation were compared using an exact test based on a Monte Carlo approach with 10000 replicates. Once allometry was filtered out, shape variation was also analyzed between sexes and populations by using the same statistical test. To filter allometry, we used the residual shape variation from the regression of log-transformed shape variables on log-transformed size.

### Genetics

DNA extraction was carried out with the Qiagen DNeasy Tissue Kit (Qiagen, Inc.) as per the manufacturer's protocol. Samples from previous field trips [3], [13], [14] were combined with the newly collected samples for the genetic analysis. The newly sampled individuals were screened for variation at nine microsatellite loci as in [13], except that the Gal263 locus was not included in the analysis. These samples were genotyped on an ABI 3730 DNA Analyzer and analyzed using both GENEMARKER 1.6 (SoftGenetics, State College, PA) and GENEMAPPER 4.0 (Applied Biosystems, Foster City, CA). Allelic richness across all microsatellite loci, observed (H_O_) and expected (H_E_) heterozygosity values were calculated using ARLEQUIN v3.11 [49]. Departure from Hardy-Weinberg equilibrium was assessed using a modification of the Markov-chain random walk algorithm described by [50] as implemented in ARLEQUIN with a Markov chain length of 1,000,000 and 100,000 burn-in steps. In addition, exact tests for heterozygote deficiency and excess were conducted using a modification of Markov chain randomization method when more than five alleles were detected per locus (with 1,000 batches with 10,000 iterations per batch, after10,000 burn-in steps) as implemented in GENEPOP v4.0.7 [51]. In all other cases exact significance values were calculated with the same software by complete enumeration [52]. Pairwise linkage disequilibrium between each pair of loci (36 pairwise comparisons) within each population was tested using a Markov chain method (100,000 burn-in steps and 1,000 batches at 10,000 iterations per batch) as implemented in GENEPOP. Multiple-test corrections were applied to Hardy-Weinberg and linkage disequilibrium tests using the sequential Bonferroni [53] correction procedures in order to control type I and type II errors. Genetic divergence was quantified in ARLEQUIN using the F_ST_ index [54] estimated by θ [55]. The program STRUCTURE 2.2 [56] was used for inferring population structure and to estimate possible admixture. This program accounts for deviations from Hardy-Weinberg and linkage disequilibrium by population structure and assigns individuals to *K* clusters. The algorithm implemented in the program uses a Bayesian approach with a Markov Chain Monte Carlo (MCMC) procedure. In our study, we evaluated prior models with *K* between 1 and 4, allowing admixture between populations and correlated allele frequencies. For each value of *K*, the MCMC was estimated after a burn-in of 100,000 steps, and a chain length of 1,000,000 steps.

To amplify the mtDNA control region, we used primers CytoR4 and DL3Rev and the PCR protocol described in [14]. Both strands were sequenced using BigDye v3.1 terminator on an ABI 3730 DNA Analyzer. Mitochondrial DNA sequences were assembled and edited with SEQUENCHER 4.2.2 (Gene Codes Corp.) and aligned in MEGA 4 [57] using CLUSTALW (GenBank accession numbers GQ259489-GQ259587). Sequences were collapsed and haplotypes were identified using COLLAPSE 1.2 [58]. Haplotype networks were constructed using the median joining method [59] in NETWORK 4.2.0.1 (Fluxus Technology Ltd.). ARLEQUIN was also used to calculate haplotype diversity, run an AMOVA (Analysis of Molecular Variance), and to quantify F_ST_ via the level of genetic divergence between the two samples (based on the Tamura and Nei [60] genetic distance).

## Supporting Information

Figure S1 Haplotype networks of the La Reserva and Cerro Fatal populations based on a fragment of the mtDNA control region. Dot size corresponds to the number of individuals sharing the same haplotype. On the bottom left, scale size is indicated. Black dots represent median vectors; each line represents one mutational step, unless recorded otherwise noted by numbers. Different colors are used to represent tortoises sampled in La Reserva (grey) and Cerro Fatal (white) (as in Figure 2).
(0.27 MB EPS)

Supporting Information S1 Measurements description
(0.05 MB DOC)
