# Supplementary material for: Morphometrics Parallel Genetics in a Newly Discovered and Endangered Taxon of Galápagos Tortoise
Source: PLoS One. 2009 Jul 17;4(7):e6272. doi: 10.1371/journal.pone.0006272 (PMC2707613; doi:10.1371/journal.pone.0006272)
Supplement: Supporting Information S1 — Measurements description (0.05 MB DOC) [file pone.0006272.s002.doc]

**Supporting information S2.** Measurements description

| **Abbreviation** | **Variable’ name** | **Description** | **Type of measurement** | **Measurement instrument** |
| --- | --- | --- | --- | --- |
| FH | Frontal height | Measurement to nuchal notch from level substrate | straight-line | caliper |
| MH | Medial height | Measurement of height of carapace | straight-line | caliper |
| H1M | Height of first marginals | Measurement to margin of first marginals from level substrate | straight-line | caliper |
| H2M | Height of second marginals | Measurement to margin of second marginals from level substrate | straight-line | caliper |
| L | Length | Length of carapace along midline | straight-line | caliper |
| CL | Curved length | Curved measurement of length of carapace along midline | curved | tape measure |
| W | Width | Width of carapace | straight-line | caliper |
| CW | Curved width | Curved measurement of width of carapace | curved | tape measure |
| W1M | Width of first marginals | Width of the first pair of marginals | straight-line | caliper |
| W2M | Width of second marginals | Width of the second pair of marginals | straight-line | caliper |
| W3M | Width of third marginals | Width of the third pair of marginals | straight-line | caliper |
| L1N | Length of first neural | Length of first neural scute on midline | curved | tape measure |
| L2N | Length of second neural | Length of second neural scute on midline | curved | tape measure |
| L3N | Length of third neural | Length of third neural scute on midline | curved | tape measure |
| L4N | Length of fourth neural | Length of fourth neural scute on midline | curved | tape measure |
| W1N | Width of first neural | Width of first neural measured from widest point | curved | tape measure |
| W2N | Width of second neural | Width of second neural measured from widest point | curved | tape measure |
| W3N | Width of third neural | Width of third neural measured from widest point | curved | tape measure |
| SW1N | Straight width of first neural | Straight width of first neural measured from widest point | straight-line | caliper |
| SW2N | Straight width of second neural | Straight width of second neural measured from widest point | straight-line | caliper |
| SW3N | Straight width of third neural | Straight width of third neural measured from widest point | straight-line | caliper |
| PL | Plastron | Length measurement along midline of plastron | straight-line | caliper |
| LG | Length of gulars | Length of gulars measured on midline | straight-line | caliper |
| LGH | Length of gulars and humerals | Length of gulars and humerals measured on midline | straight-line | caliper |
| WH | Width of humerals | Width of second pair of scutes on plastron measured from widest point | straight-line | caliper |
| AL | Length of anals | Length of anal scutes measured on midline | straight-line | caliper |
